# Supplementary material for: Additive and interactive effects of spatial attention and expectation on perceptual decisions
Source: Sci Rep. 2018 Apr 30;8:6732. doi: 10.1038/s41598-018-24703-6 (PMC5928039; doi:10.1038/s41598-018-24703-6)
Supplement: Supplementary file 1 — Supplementary Information [file 41598_2018_24703_MOESM1_ESM.pdf]

Additive and interactive effects of spatial attention and expectation on perceptual decisions

Authors: Arianna Zuanazzi[1], Uta Noppeney[1]

[1]Computational Cognitive Neuroimaging Laboratory, Computational Neuroscience and Cognitive Robotics Centre, University of Birmingham, B15 2TT Birmingham, UK

Supplementary information

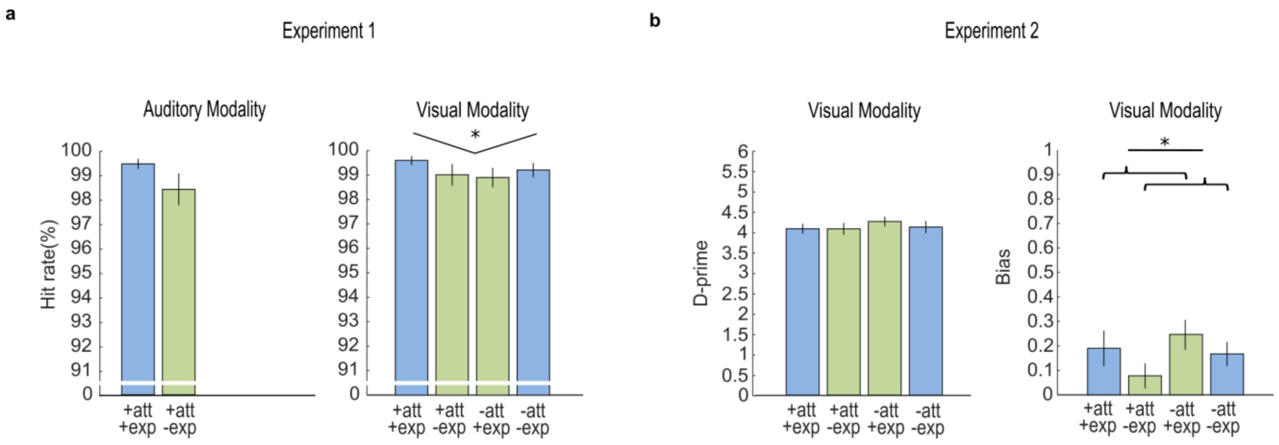

Figure S1 | Experiment 1: hit rates; Experiment 2: d-prime and bias

a. Experiment 1: hit rates for each of the six conditions with response requirements. The brackets and stars indicate significance of main effects and interactions. * $p < 0.05$.

b. Experiment 2: d-prime and bias values for each of the four conditions for the visual modality. The brackets and stars indicate significance of main effects and interactions. * $p < 0.05$.

Table S1. Spatial signal probability, general response probability and spatially selective response probability for each condition in experiment 1 and experiment 2.

S = signal location, $H_{..att..exp}$ = hemifield e.g. +att +exp, R = response.

	Conditions			
	+att +exp	+att -exp	-att +exp	-att -exp
Experiment 1				
Spatial signal probability $P(S = H_{..att..exp})$	0.65	0.35	0.65	0.35
General response probability $P(R = +)$	0.9	0.6	0.6	0.9
Spatially selective response probability $P(R = + \mid S = H_{..att..exp})$	1	1	0.384	0.714
Experiment 2				
Spatial signal probability $P(S = H_{..att..exp})$	0.625	0.375	0.625	0.375
General response probability $P(R = +)$	0.123	0.123	0.123	0.123
Spatially selective response probability $P(R = + \mid S = H_{..att..exp})$	0.131	0.218	0.065	0.109

Please note that these probabilities are computed pooled over auditory and visual modalities.

The spatial signal probability is the probability that a signal is presented in a particular hemifield, e.g. in experiment 1: P = 0.65 for the hemifield that is attended and expected vs. P = 0.35 for the hemifield that is unattended and unexpected (n.b. the spatial signal probabilities sum to one for the two conditions presented in the same run).

The general response probability is the probability that a trial requires a response in a particular run irrespective of where the signal is presented, e.g. in experiment 1: $P = 0.9$ for runs where attention and expectation are congruent vs. $P = 0.6$ for runs where attention and expectation are incongruent.

The spatially selective response probability is the probability that a response is required conditioned on the signal being presented in a particular hemifield, e.g. in experiment 1: the probability of a response is $P = 0.384$, if the signal is presented in a hemifield that is not attended but expected.

As a result, we can compute the general response probability by multiplying the spatially selective response probability with the spatial signal probability and marginalizing over the spatial signal probability; e.g.

$$P(R = +) = P(R = +|S = H_{+att+exp})\, P(S = H_{+att+exp}) + P(R = +|S = H_{-att-exp})\, P(S = H_{-att-exp})$$
